# Supplementary material for: Co-contraction embodies uncertainty: An optimal feedforward strategy for robust motor control
Source: PLoS Comput Biol. 2024 Nov 20;20(11):e1012598. doi: 10.1371/journal.pcbi.1012598 (PMC11616891; doi:10.1371/journal.pcbi.1012598)
Supplement: S1 Text — (PDF) [file pcbi.1012598.s002.pdf]

Supporting Information Text

Co-Contraction Embodies Uncertainty: An Optimal Feedforward Strategy for Robust Motor Control

Bastien Berret, Dorian Verdel, Etienne Burdet, and Frédéric Jean

In this Supporting Information Text, we first give details about the case of a discrete random variable which introduces an alternative method of resolution. We then provide the proofs related to the analytical toy example and Proposition 1 of the main text.

Case of a discrete random variable $\boldsymbol{\xi}$

When $\boldsymbol{\xi}$ is a discrete random variable, we can address the problem differently. Let us assume that the random variable can take the values $\boldsymbol{\xi}_i$ for $i = 1, ..., s$ with probability α_i, and we denote $\mathbf{x}^{\boldsymbol{\xi}_i}(\cdot)$ the n-dimensional trajectory associated with the control $\mathbf{u}(\cdot)$ and the value $\boldsymbol{\xi}_i$. Given the nature of the problem, the expected cost in Eq. 2 of the main text can be rewritten

$$J(\mathbf{u}) = \sum_{i=1}^{s} \alpha_i q_f(\mathbf{x}^{\boldsymbol{\xi}_i}(T), T) + \int_0^T \sum_{i=1}^{s} \alpha_i q(\mathbf{x}^{\boldsymbol{\xi}_i}(t), \mathbf{u}(t), t)\, dt, \tag{1}$$

where q_f and q are quadratic functions in the state.

If we augment the state of the system by setting $\tilde{\mathbf{x}} = (\mathbf{x}^{\boldsymbol{\xi}_1}, \ldots, \mathbf{x}^{\boldsymbol{\xi}_s})$, the cost can be written more compactly as

$$J(\mathbf{u}) = \tilde{q}_f(\tilde{\mathbf{x}}(T), T) + \int_0^T \tilde{q}(\tilde{\mathbf{x}}(t), \mathbf{u}(t), t)\, dt, \tag{2}$$

where the function $\tilde{q}(\tilde{\mathbf{x}}(t), \mathbf{u}(t), t) = \sum_{i=1}^{s} \alpha_i q(\mathbf{x}^{\boldsymbol{\xi}_i}(t), \mathbf{u}(t), t)$ and $\tilde{q}_f(\tilde{\mathbf{x}}(T), T) = \sum_{i=1}^{s} \alpha_i q_f(\mathbf{x}^{\boldsymbol{\xi}_i}(T), T)$.

The initial uncertain optimal control problem with discrete random uncertainty $\boldsymbol{\xi}$ is equivalent to a deterministic optimal control problem in the augmented state $\tilde{\mathbf{x}} \in \mathbb{R}^{ns}$ with a dynamical system of the form

$$\dot{\tilde{\mathbf{x}}}(t) = \tilde{\mathbf{f}}(\tilde{\mathbf{x}}(t), \mathbf{u}(t), t) \tag{3}$$

where $\tilde{\mathbf{f}} = (\mathbf{f}(\mathbf{x}^{\boldsymbol{\xi}_1}(t), \mathbf{u}(t), t; \boldsymbol{\xi}_1), \ldots, \mathbf{f}(\mathbf{x}^{\boldsymbol{\xi}_s}(t), \mathbf{u}(t), t; \boldsymbol{\xi}_s))$.

Remark. There is no restriction on the type of distribution for $\boldsymbol{\xi}$ and on the type of dynamics in this approach. The main limitation is the size of the augmented space (ns), which may limit the relevance of the approach when s is too large. In this case, the approach proposed in Problem 3 of the main text may be more suited. If the system is governed by the nonlinear SDE

$$d\mathbf{x}_t = \mathbf{f}(\mathbf{x}_t, \mathbf{u}(t), t; \boldsymbol{\xi})\, dt + \mathbf{G}(\mathbf{x}_t, \mathbf{u}(t), t)\, d\mathbf{w}_t$$

where $\boldsymbol{\xi}$ a discrete random variable, the above approach can be combined with the SOOC approach of [1, 2], that is, we can introduce the variables $\mathbf{m}^{\boldsymbol{\xi}_i}(t)$ and $\mathbf{P}^{\boldsymbol{\xi}_i}(t)$ for $i = 1..s$.

Proof of the toy stabilization task

To illustrate that random external disturbances lead to impedance control, let us consider a toy stabilization task where analytical computations are tractable. We consider the bilinear system

$$\dot{x}(t) = f(t) - k(t)x(t) + \xi, \tag{4}$$

where x is the scalar state, $\mathbf{u} = [f, k]^\top$ is the control vector composed of a term f representing a force and a term k representing a stiffness, and ξ is a random external disturbance.

Here the random variable ξ corresponds to an external force that can be applied or not, so that ξ equals 0 with probability α and 1 with probability $1 - \alpha$.

We assume as previously that the control law $\mathbf{u}(t)$ is open-loop, and is determined as the one that minimizes the expected cost with scalar weights $q \geq 1$, $q_f > 0$,

$$J(\mathbf{u}) = \mathbb{E}\left[q_f x(T)^2 + \int_0^T \left(f(t)^2 + k(t)^2 + qx(t)^2\right)\, dt\right],$$

among the *open-loop* controls $\mathbf{u}(t)$ ensuring that the expectation $\mathbb{E}_\xi[x(T)]$ of the final state equals 0. We assume that the initial state is $x(0) = 0$ and that time T is fixed.

We will now prove that the very presence of uncertainty leads to impedance control in this problem.

First case (without uncertainty). Consider the cases where there is no uncertainty, that is when $\alpha = 0$ or $\alpha = 1$. It consists in minimizing the cost

$$J(\mathbf{u}) = q_f x(T)^2 + \int_0^T \left(f(t)^2 + k(t)^2 + qx(t)^2\right)\, dt,$$

among the trajectories $(x, \mathbf{u})$ of the deterministic controlled system

$$\dot{x}(t) = f(t) - k(t)x(t) + \alpha,$$

satisfying $x(0) = x(T) = 0$.

Let us apply the necessary conditions of the Pontryagin's Maximum Principle (PMP) (see [3]). The Hamiltonian function is

$$\mathcal{H}(x, \lambda, \mathbf{u}) = \lambda(f - kx + \alpha) - \frac{1}{2}\left(f^2 + k^2 + qx^2\right).$$

On the one hand, each value of the optimal control $\mathbf{u}$ must maximize $\mathcal{H}$ with respect to the control, which yields $f = \lambda, k = -\lambda x$. On the other hand, the optimal trajectory and its coadjoint variable λ must satisfy the Hamiltonian differential equations

$$\dot{x} = \lambda(1 + x^2) + \alpha, \qquad \dot{\lambda} = x(q - \lambda^2).$$

Taking the derivative of $\dot{x}$, we obtain

$$\ddot{x} = \begin{cases} x(1 + x^2)(q + \lambda^2) & \text{if } \alpha = 0, \\ x\left(x^2(q + \lambda^2) + (1 + \lambda)^2 + q - 1\right) & \text{if } \alpha = 1. \end{cases}$$

Thus $x\ddot{x} \geq 0$ (recall $q \geq 1$) which implies that the function $t \mapsto (x\dot{x})(t)$ is nondecreasing. Since this function is zero at $t = 0$, it is nonnegative, therefore $x^2(t)$ is nondecreasing. The condition $x(0) = x(T) = 0$ then implies that the optimal trajectory is $x \equiv 0$ and the optimal control satisfies $f \equiv -\alpha, k \equiv 0$, showing that there is no use of stiffness (or impedance control) in these cases.

Second case (with uncertainty). Let us now show that the above situation ($k \equiv 0$) never appears in the presence of uncertainty, that is, when $0 < \alpha < 1$.

We first convert the optimal control problem into a deterministic one by augmenting the state of the system. We set $\mathbf{x} = (x_0, x_1)$, with an augmented dynamics

$$\dot{x}_0(t) = f(t) - k(t)x_0(t), \qquad \dot{x}_1(t) = f(t) - k(t)x_1(t) + 1, \tag{5}$$

initial conditions $\mathbf{x}(0) = 0$, and a terminal condition $\mathbb{E}_\xi[x(T)] = (1 - \alpha)x_0(T) + \alpha x_1(T) = 0$. The cost writes as

$$J(\mathbf{u}) = q_f((1 - \alpha)x_0(T)^2 + \alpha x_1(T)^2) + \int_0^T \left(f(t)^2 + k(t)^2 + q(1 - p)x_0(t)^2 + qpx_1(t)^2\right) dt.$$

The solutions of the corresponding optimal control problem must satisfy the necessary condition given by the PMP. Define the Hamiltonian function

$$\mathcal{H}(\mathbf{x}, \boldsymbol{\lambda}, \mathbf{u}, \nu) = \lambda_0(f - kx_0) + \lambda_1(f - kx_1 + 1) - \nu\left(f^2 + k^2 + q(1 - \alpha)x_0^2 + q\alpha x_1^2\right). \tag{6}$$

If $\mathbf{u}(t)$ is an optimal control with associated trajectory $\mathbf{x}(t)$, then there exist $\nu = 0$ or $1/2$ and a function $\boldsymbol{\lambda}(t) = (\lambda_0(t), \lambda_1(t))$ such that $(\nu, \boldsymbol{\lambda}) \neq 0$ and, for every $t \in [0, T]$,

$$\dot{\lambda}_0 = -\frac{\partial \mathcal{H}}{\partial x_0}(\mathbf{x}, \boldsymbol{\lambda}, \mathbf{u}, \nu), \quad \dot{\lambda}_1 = -\frac{\partial \mathcal{H}}{\partial x_1}(\mathbf{x}, \boldsymbol{\lambda}, \mathbf{u}, \nu), \tag{7}$$

$$\mathcal{H}(\mathbf{x}(t), \boldsymbol{\lambda}(t), \mathbf{u}(t), \nu) = \max_{\mathbf{v}} \mathcal{H}(\mathbf{x}(t), \boldsymbol{\lambda}(t), \mathbf{v}, \nu), \tag{8}$$

plus conditions of transversality that we do not need here. A simple computation shows that $\nu = 0$ leads to a contradiction, so $\nu = 1/2$ and the maximization condition on $\mathcal{H}$ is equivalent to

$$f = \lambda_0 + \lambda_1, \qquad k = -\lambda_0 x_0 - \lambda_1 x_1, \tag{9}$$

whereas the Hamiltonian differential equations write as

$$\dot{\lambda}_0 = q(1-\alpha)x_0 + k\lambda_0, \qquad \dot{\lambda}_1 = q\alpha x_1 + k\lambda_1. \tag{10}$$

Assume by contradiction that there exists an optimal solution with no stiffness, that is, an optimal control with $k \equiv 0$. This implies by Eqs. 5, 9 and 10 that there exist a solution $(\mathbf{x}(t), \boldsymbol{\lambda}(t))$ of

$$\left\{ \begin{array}{l} \dot{x}_0 = \lambda_0 + \lambda_1, \\ \dot{x}_1 = \lambda_0 + \lambda_1 + 1, \end{array} \right. \qquad x_0(0) = x_1(0) = 0, \qquad \left\{ \begin{array}{l} \dot{\lambda}_0 = q(1-\alpha)x_0, \\ \dot{\lambda}_1 = q\alpha x_1, \end{array} \right. \tag{11}$$

verifying $k = -\lambda_0 x_0 - \lambda_1 x_1 \equiv 0$. A simple computation shows that the solutions of the above differential equations are of the form

$$\left\{ \begin{array}{l} x_0 = -\alpha t + a\sinh(\sqrt{q}t), \\ x_1 = (1-\alpha)t + a\sinh(\sqrt{q}t), \end{array} \right. \qquad \left\{ \begin{array}{l} \lambda_0 = b_0 - q\frac{\alpha(1-\alpha)}{2}t^2 + a\sqrt{q}(1-\alpha)\cosh(\sqrt{q}t), \\ \lambda_1 = b_1 + q\frac{\alpha(1-\alpha)}{2}t^2 + a\sqrt{q}\alpha\cosh(\sqrt{q}t), \end{array} \right. \tag{12}$$

for some constants a, b_0, b_1. As a consequence, $k = -\lambda_0 x_0 - \lambda_1 x_1$ writes as

$$k = (\alpha b_0 - (1-\alpha)b_1)\, t - q\frac{\alpha(1-\alpha)}{2}t^3 + a(b_0 + b_1)\sinh(\sqrt{q}t) - a^2\sqrt{q}\sinh(\sqrt{q}t)\cosh(\sqrt{q}t), \tag{13}$$

and cannot be identically zero when $0 < \alpha < 1$, whatever the values of the constants a, b_0, b_1.

We thus get the conclusion that uncertainty ($0 < \alpha < 1$) leads to some nonzero impedance control ($k \not\equiv 0$). Further note that the random variables has mean α and variance $\alpha(1-\alpha)$ so that it can be seen that the level of stiffness directly depends on the variance (that is, the degree of task uncertainty).

Proof of Proposition 1 of the main text

The Proposition results from the two following lemmas.

Lemma 1. *Assume that the dynamics* $\mathbf{f}$ *are smooth with compact support and depend affinely on the*

random parameter $\boldsymbol{\xi}$*, i.e.*

$$\mathbf{f}(\mathbf{x}, \mathbf{u}; \boldsymbol{\xi}) = \mathbf{g}(\mathbf{x}, \mathbf{u}) + \mathbf{H}(\mathbf{u})\boldsymbol{\xi}.$$

Then there exists a constant $C > 0$ *such that, for any* $t \in [0, T]$*,*

$$\sup_{s\in[0,t]} \|\mathbf{m_x}(s) - \mathbf{m}(s)\|^2 + \sup_{s\in[0,t]} \|\mathbf{P_x}(s) - \mathbf{P}(s)\| \leq C \left(\sup_{s\in[0,t]} \|\mathbf{P}(s)\| + \sup_{s\in[0,t]} \|\mathbf{D}(s)\| \right).$$

Proof. Note first that, by a direct computation, $\mathbf{m_x} = \mathbb{E}[\mathbf{x}]$, $\mathbf{P_x} = \mathbb{E}[\big(\mathbf{x} - \mathbf{m_x}\big)\big(\mathbf{x} - \mathbf{m_x}\big)^\top]$ and $\mathbf{D_x} = \mathbb{E}[\big(\mathbf{x} - \mathbf{m_x}\big)\big(\boldsymbol{\xi} - \boldsymbol{\mu}\big)^\top]$ satisfy

$$\begin{aligned}
\dot{\mathbf{m}}_{\mathbf{x}} = \quad & \mathbb{E}[\mathbf{g}(\mathbf{x}, \mathbf{u})] + \mathbf{H}(\mathbf{u})\boldsymbol{\mu}, \\
\dot{\mathbf{D}}_{\mathbf{x}} = \quad & \mathbb{E}\left[\big(\mathbf{g}(\mathbf{x}, \mathbf{u}) - \mathbb{E}[\mathbf{g}(\mathbf{x}, \mathbf{u})]\big)\big(\boldsymbol{\xi} - \boldsymbol{\mu}\big)^\top\right] + \mathbf{H}(\mathbf{u})\boldsymbol{\Sigma}, \\
\dot{\mathbf{P}}_{\mathbf{x}} = \quad & \mathbb{E}\left[\big(\mathbf{g}(\mathbf{x}, \mathbf{u}) - \mathbb{E}[\mathbf{g}(\mathbf{x}, \mathbf{u})]\big)\big(\mathbf{x} - \mathbf{m_x}\big)^\top\right] + \mathbb{E}\left[\big(\mathbf{x} - \mathbf{m_x}\big)\big(\mathbf{g}(\mathbf{x}, \mathbf{u}) - \mathbb{E}[\mathbf{g}(\mathbf{x}, \mathbf{u})]\big)^\top\right] \\
& + \mathbf{H}(\mathbf{u})\mathbf{D}_{\mathbf{x}}^\top + \mathbf{D_x}\mathbf{H}(\mathbf{u})^\top.
\end{aligned} \tag{14}$$

whereas $(\mathbf{m}, \mathbf{P}, \mathbf{D})$ satisfies Eq. 18 of the main text, and $(\mathbf{m_x}, \mathbf{P_x}, \mathbf{D_x})(0) = (\mathbf{m}, \mathbf{P}, \mathbf{D})(0)$.

We will use a Taylor expansion with integral rest of the function $\mathbf{g}$,

$$\mathbf{g}(\mathbf{x}, \mathbf{u}) = \mathbf{g}(\mathbf{m}, \mathbf{u}) + \mathbf{h}(\mathbf{m}, \mathbf{x}, \mathbf{u})(\mathbf{x} - \mathbf{m}),$$

the function $\mathbf{h}$ being smooth with compact support. Thus,

$$\|\dot{\mathbf{m}}_{\mathbf{x}} - \dot{\mathbf{m}}\| \leq \|\mathbb{E}\left[\mathbf{g}(\mathbf{x}, \mathbf{u})\right] - \mathbf{g}(\mathbf{m}, \mathbf{u})\| \leq \|\mathbb{E}\left[\mathbf{h}(\mathbf{m}, \mathbf{x}, \mathbf{u})(\mathbf{x} - \mathbf{m})\right]\| \leq C\|\mathbb{E}\left[\mathbf{x} - \mathbf{m}\right]\|$$

This inequality, together with Cauchy-Schwarz and Jensen inequalities, yields to

$$\begin{aligned}
\sup_{s\in[0,t]} \|\mathbf{m_x}(s) - \mathbf{m}(s)\|^2 &\leq \left(\int_0^t \|\dot{\mathbf{m}}_{\mathbf{x}}(s) - \dot{\mathbf{m}}(s)\| \, ds\right)^2 \\
&\leq C \left(\int_0^t \|\mathbb{E}\left[\mathbf{x}(s) - \mathbf{m}(s)\right]\| \, ds\right)^2 \\
&\leq C \int_0^t \|\mathbb{E}\left[\mathbf{x}(s) - \mathbf{m}(s)\right]\|^2 \, ds.
\end{aligned}$$

Writing $\mathbf{x} - \mathbf{m}$ as $\mathbf{x} - \mathbf{m_x} + \mathbf{m_x} - \mathbf{m}$ and using the fact that $\mathrm{tr}(\mathbf{P_x}) \leq C\|\mathbf{P_x}\|$, we obtain

$$\begin{aligned}
\sup_{s\in[0,t]} \|\mathbf{m_x}(s) - \mathbf{m}(s)\|^2 &\leq C \int_0^t \big(\|\mathbf{m_x}(s) - \mathbf{m}(s)\|^2 + \mathrm{tr}(\mathbf{P_x}(s))\big) \, ds, \\
&\leq C \left(\int_0^t \sup_{\tau\in[0,s]} \|\mathbf{m_x}(\tau) - \mathbf{m}(\tau)\|^2 \, ds + \sup_{s\in[0,t]} \|\mathbf{P_x}(s)\|\right).
\end{aligned}$$

Finally, using Gronwall's inequality and $\mathbf{P_x} = \mathbf{P_x} - \mathbf{P} + \mathbf{P}$, we obtain

$$\sup_{s\in[0,t]} \|\mathbf{m_x}(s) - \mathbf{m}(s)\|^2 \leq C \left(\sup_{s\in[0,t]} \|\mathbf{P_x}(s) - \mathbf{P}(s)\| + \sup_{s\in[0,t]} \|\mathbf{P}(s)\| \right).$$

A similar estimate shows

$$\begin{aligned} \sup_{s\in[0,t]} \|\mathbf{P_x}(s) - \mathbf{P}(s)\| \leq & \quad C \left(\sup_{s\in[0,t]} \|\mathbf{D_x}(s) - \mathbf{D}(s)\| + \sup_{s\in[0,t]} \|\mathbf{P}(s)\| + \sup_{s\in[0,t]} \|\mathbf{D}(s)\| \right), \\ \sup_{s\in[0,t]} \|\mathbf{D_x}(s) - \mathbf{D}(s)\| \leq & \quad C \sup_{s\in[0,t]} \|\mathbf{D}(s)\|. \end{aligned}$$

Combining the last three inequalities, we obtain the lemma. □

Lemma 2. *Assume that the dynamics* $\mathbf{f}$ *is smooth with compact support. Then there exists a constant* $C > 0$ *such that, for any* $t \in [0, T]$,

$$\sup_{s\in[0,t]} \|\mathbf{m_x}(s) - \mathbf{m}(s)\|^2 + \sup_{s\in[0,t]} \|\mathbf{P_x}(s) - \mathbf{P}(s)\| \leq C \left(\sup_{s\in[0,t]} \|\mathbf{P}(s)\| + \sup_{s\in[0,t]} \|\mathbf{D}(s)\| + \|\mathbf{\Sigma}\| \right).$$

Proof. Set $\widetilde{\mathbf{x}} = (\mathbf{x}, \boldsymbol{\xi})$, $\widetilde{\boldsymbol{\xi}} = \mathbf{0}$, $\widetilde{\mathbf{x}}_0 = (\mathbf{x}_0, \boldsymbol{\xi})$, and

$$\widetilde{\mathbf{f}}(\widetilde{\mathbf{x}}, \mathbf{u}; \widetilde{\boldsymbol{\xi}}) = \begin{pmatrix} \mathbf{f}(\mathbf{x}, \mathbf{u}; \boldsymbol{\xi}) \\ \mathbf{0} \end{pmatrix}.$$

With the notations of the Materials and Methods, we have for the extended system:

$$\widetilde{\mathbf{m}}_\mathbf{x} = (\mathbf{m_x}, \boldsymbol{\mu}), \quad \widetilde{\mathbf{m}} = (\mathbf{m}, \boldsymbol{\mu}), \quad \widetilde{\mathbf{D}}_\mathbf{x} = \widetilde{\mathbf{D}} = \mathbf{0}, \quad \widetilde{\mathbf{P}}_\mathbf{x} = \begin{pmatrix} \mathbf{P_x} & \mathbf{D_x} \\ \mathbf{D_x}^\top & \mathbf{\Sigma} \end{pmatrix}, \quad \widetilde{\mathbf{P}} = \begin{pmatrix} \mathbf{P} & \mathbf{D} \\ \mathbf{D}^\top & \mathbf{\Sigma} \end{pmatrix}.$$

Since the dynamics $\widetilde{\mathbf{f}}$ is not perturbed by a random parameter, it can be considered as affine with respect to this parameter. Thus Lemma 1 applies and we obtain the desired estimate. □

References

1. Berret B, Jean F. Efficient computation of optimal open-loop controls for stochastic systems. Automatica. 2020;115:108874. doi:https://doi.org/10.1016/j.automatica.2020.108874.

2. Berret B, Jean F. Stochastic optimal open-loop control as a theory of force and impedance planning via muscle co-contraction. PLOS Computational Biology. 2020;16(2):e1007414. doi:10.1371/journal.pcbi.1007414.

3. Trélat E. Contrôle optimal : Théorie & applications. Vuibert, editor; 2008.
