# Supplementary material for: Co-contraction embodies uncertainty: An optimal feedforward strategy for robust motor control
Source: PLoS Comput Biol. 2024 Nov 20;20(11):e1012598. doi: 10.1371/journal.pcbi.1012598 (PMC11616891; doi:10.1371/journal.pcbi.1012598)
Supplement: Attachment — Submitted filename: response_letter.pdf [file pcbi.1012598.s003.pdf]

We thank the reviewers and the editor for their insightful and constructive feedback, which has helped us enhance the clarity of our paper. Below, we address each comment individually, with our responses highlighted in blue.

In summary, the key revisions include:

- Extending the explanation of the framework modeling capabilities.
- Enhancing the discussion on potential future applications of the framework.
- Providing additional details on each simulation.

Reviewer #1

This paper presents theoretical analysis and experimental evidence that feedforward modulation of muscle co-activation and mechanical impedance is a strategy to deal with uncertain external perturbations. The paper is generally well written and the experimental results are largely consistent with the model predictions. However, the experiment considered only a single-joint pointing task which is hardly a "seminal motor control task" (line 89). This significantly reduced enthusiasm for the findings reported.

We thank the reviewer for her review and comments. We chose to validate the proposed uncertain stochastic open-loop optimal control (USOOC) model using a single-joint pointing task involving wrist flexion/extension movements as this enabled us to precisely analyze and simulate muscle activity. The task was thus specifically designed to test the model's predictions with a Bernoulli type of external disturbance.

Motor control studies often focus on 1-dof systems as they enable maximum control over experimental parameters, which is crucial for an initial test of a theory. In our view, this task provided a straightforward experiment to examine the relationship between external uncertainty and impedance. However, the method is easily adaptable to multi-joint systems, as demonstrated by the 2-dof arm simulation. Higher dimensional simulations are also possible although more computationally intensive. Although the word "seminal" was also referring to the simulations of classical tasks (e.g., refs. 3 and 4), we have removed it from the quoted sentence (line 104) in the revised manuscript.

Moreover, the finding that for α of 0.25, 0.50 and 0.75, performance converged within 10 to 20 trials (figure 5C) would appear to indicate that this was not a particularly challenging task. How then can extrapolation to more general tasks be justified?

While the task involved only 1-dof, it was not straightforward to execute. The uncertainty and late timing of the disturbance presented significant challenges, making it difficult to succeed on each trial. In the 100% condition, which preceded the α={0.25, 0.50, 0.75} conditions, participants required an average of about 50 trials to adapt and achieve a success rate above 80%. After this initial adaptation, participants indeed needed approximately 20 trials to adjust to the uncertain conditions, However, it is important to note that the nature of the perturbation was known prior to these 20 trials, and the probability of occurrence of the perturbation was explicitly communicated to the participants.

Regarding the extrapolation to more general tasks, we note that other authors have also observed an increase of co-contraction while adapting to randomly triggered force fields during a 2-dof reaching task (ref. 38 in the text). This suggests that our findings could extend to other tasks as well. Here, our conclusions are drawn from the variety of simulated tasks, all of which demonstrate a consistent relationship between task uncertainty and impedance, despite variations in muscle modeling, simulation parameters, and task nature.

In response to the reviewer's comment, we have added a note in the Discussion highlighting the need for further research to verify whether similar results apply to other tasks and systems (lines 344-350):

"Additionally, while this study utilized simple models of force/impedance and muscle viscoelasticity, it could be beneficial to explore more advanced models that accurately represent the intrinsic viscoelastic properties of muscles (e.g., [74, 75]). The proposed framework should be versatile enough to accommodate these complex muscle models, provided their dimensionality remains manageable, and they involve differentiable functions. Further experimental studies with more naturalistic behaviors would be valuable for testing the theory and deepening our understanding of how humans balance feedforward impedance and feedback control [28]."

It appears (equation 1) that the external disturbance process ξ was stationary and independent of the system state. While that is an important case, it seems like a rather impoverished model of tool use, where the user's actions significantly affect the stochasticity of the interaction as well as its stability.

Thank you for this remark. In Eq. 1, ξ is a random variable that appears in the dynamics, so that its value is determined on each trial with some probability. However, this value can be multiplied by any function that depends on the state, control and time. Specifically, the external random disturbance can take the form $H(x,u,t)\xi$. Thus, while an instance of ξ is randomly sampled on each trial, the resulting external disturbance can take a more complex form. We now mention this important aspect on lines 51-53 and emphasize it lines 584 and 603-604.

Indeed, due to the apparent absence of state-dependent disturbances, it is not clear how equation (1) was applied to the simulation of figure 3. That may reflect this reviewers ignorance of the mathematics but if so, this should be clarified in the text.

The preceding comment may help clarify this point. In Figure 3, the force field is indeed state-dependent, so the drift term can be expressed as $f(x,u;\xi) = g(x,u) + H(x)\xi$ in this specific case. When ξ is a Bernoulli variable, $H(x)$ represents an external force field that occurs with probability α on each trial (see lines 566-594).

The results presented in figure 2 are interesting but they indicate an approximately trapezoidal time profile of stiffness. How can this be reconciled with the results reported by Bennett et al. (ref. 31) which clearly showed a substantial reduction of stiffness in the middle of movement? Indeed, might Bennett et al.'s results invalidate the analysis presented here? This should at least be discussed briefly.

The task in Figure 2 involves a static postural task (an inverted pendulum meant to remain vertical), while Bennett's work focused on dynamic movements. In ref. 23, we investigated

Bennett's task and observed a stiffness profile with a reduction in the middle of the movement. Therefore, there is no discrepancy.

The paper is focused on stiffness modulation but neuromuscular mechanical impedance may have other components. Was there any evidence of modulation of apparent neuromuscular damping?

While we focus in this paper on stiffness modulation, impedance indeed encompasses more than just that. The models also make prediction about damping when it can be modulated through co-contraction. In our models, stiffness and damping largely co-vary, making the inclusion of damping redundant in this context. We have noted this point on lines 243-244.

I would like to know more about the exoskeleton. How "back-drivable" was it? For example, if its static friction or apparent inertia were significant (which is typical for a geared electric motor) they would evoke enhanced muscle activation and thereby enhance apparent mechanical impedance with no relation to external perturbations.

We thank the reviewer for noting this point. One of the advantages of the HRX-1 is its mechanical simplicity: it does not include any gear or specific transmission, allowing the user to interact directly with the motor axis. Although a torque sensor is placed between the handle and the motor's output, this does not significantly impact effort transmission. The motor axis being mounted on bearings, the apparent inertia and the friction are negligible. A shortcoming of this interface is its relatively low maximum torque of 2Nm, which may limit its use in experiments involving joints other than the wrist. We have improved the HRX-1 description at lines 475-481.

Minor suggestions

The use of the symbol u to represent either (i) generic control input (ii) torque and (iii) stiffness (e.g. in figure 2) was quite confusing; torque and stiffness require different units. It would be helpful to distinguish these different meanings of this variable.

Thanks for bringing this to our attention. We have revised the figure and provided a clearer description of the underlying simulation in the Methods section, ensuring that the different meanings of the various variables depending on u are now distinguished. This should clarify the meanings and units of all variables.

Abstract line 7, Line 108, Line 268: Do you mean "singular" in the mathematical sense or do you mean "unique"? If the former, please clarify.
For clarity we replaced "singular" by "characteristic".

Line 13: re-arrange the sentence to read: "… predict co-contraction well …"
Thank you. We have corrected as suggested.

Line 21: while direct measurement of output mechanical impedance requires external perturbation, it may also be estimated from a model. Remarkably, there is evidence that humans do so [1],[2]. This might support the argument for predictive feedforward control of mechanical impedance.

Thank you for pointing out this connection. We have now cited these papers in the Introduction to support our hypothesis on predictive feedforward control of mechanical impedance, at lines 20-22.

In equation (1) should the symbol be \w_t or w as defined in the subsequent text?

We have clarified the definition of the Wiener process at line 49.

Line 259: delete "of"
Line 236: suggest replacing "At last" with "Finally".
Line 454: rearrange the sentence to read: "… the considered reaching movement."

Thank you for these suggestions, which we have adopted in the revised manuscript.

References
[1] M. E. Huber, C. Folinus, and N. Hogan, "Visual Perception of Joint Stiffness from Multi-Joint Motion," J. Neurophysiol., vol. 122, no. 1, pp. 51–59, 2019, doi: 10.1152/jn.00514.2018.

[2] A. M. J. West, M. E. Huber, and N. Hogan, "Role of Path Information in Visual Perception of Joint Stiffness," PLoS Comput. Biol., vol. 18, no. 11, p. e1010729, 2022, doi: 10.1371/journal.pcbi.1010729.

Reviewer #2

This paper presents a new mathematical framework to explain human sensorimotor behavior in the face of environmental uncertainty. The framework and the experimental evaluations focus on feedforward (open-loop) control, showing that muscle co-contraction is related to environmental uncertainty. The results provide an insightful first step towards understanding why and how co-contraction occurs, and can be a valuable addition to the literature.

However, I have some general thoughts that the authors might consider addressing to further strengthen the paper. One point for further consideration is the potentially restrictive formulation of the problem. The authors model "trial-by-trial" uncertainty, where the uncertain effects are fixed within the scope of one trial (encoded in ξ), but we live in a continuous world; trial-by-trial effects are artifacts of our experimental designs. I recognize the limitations of our current experimental and theoretical tools, and that answering such real-world questions will require decades of research. Nevertheless, I encourage the authors to discuss this issue further. I personally would be very interested to learn their perspective on how these results might be extended to more real-world applications, such as implementing the framework within a receding horizon control scheme.

Thank you for the insightful comment. Implementing the framework within a receding horizon control scheme is indeed feasible and something we envision for future work. In this case, the random disturbance would simply occur with a given probability in the planning horizon, eliminating the trial-by-trial context. The USOOC model would operate continuously, with the probability α set at the beginning of each time horizon where planning occurs. While ξ could become implicitly time-varying as it could be changed at each time step of the control loop, it would remain a random parameter to account for to plan

the current motor command. Therefore, the proposed framework should be applicable in a receding horizon context, and the level of uncertainty would influence the resulting motor command. In this revision, we have emphasized that more uncertainty about the initial state readily yields more co-contraction or stiffness at the beginning of the time horizon (e.g., see Figure 1 and below). As only the first value of the control is commonly used in a receding horizon control scheme, it should thus predict to exploit co-contraction/impedance in this context if the state estimate is uncertain. We added a discussion on this matter at lines 319-327, which could be important for future developments: *"The second one is to consider a model predictive control (MPC) approach [64–66]. Intermittent feedback control could allow the system to re-estimate its current state and plan a feedforward motor command on a receding horizon [67]. In particular, if the current state estimate has large covariance, our model will immediately command a large stiffness or co-contraction (e.g., see Fig. 1). In practice the MPC framework could be interesting to go beyond the trial-by-trial context of in-lab experiments. In that case, the disturbance ξ would just encode the uncertainty of a given disturbance in the subsequent planning horizon. In any case, the cornerstone of high-level feedback control is the state estimation process optimally mixing sensory information and predictions with forward models [...]"*

My second comment is regarding the level of detail. The paper presents the general problem that the nervous system may need to solve (eqs. 1 and 2) and then converts the original problem to an equivalent ODE problem. However, the final step—solving the problem—is not fully detailed. Depending on the reader's goal—whether learning about the functioning of the brain or implementing/extending the method—the level of detail might be either too much or too little. Adjusting the level of detail to better match the overall objective of the paper would be beneficial.

Thank you for pointing this out. We have adjusted the level of detail accordingly. First, we have enhanced the descriptions of each simulation to also align with some comments from Reviewer 1. Second, we retained all relevant mathematical derivations to facilitate implementation by readers, such as for developing a receding horizon control scheme in their preferred programming language. Finally, we provide fewer specifics on the exact implementation because (i) there are various software options for solving deterministic optimal control problems and (ii) we have included the code used for each example. Interested readers can examine our code, which is relatively straightforward to understand due to the close alignment of Julia syntax with mathematics notation. Please see the additions to the description of the simulations at lines 548 to 594.

Another philosophical point concerns the paper's conclusion: "Co-contraction embodies uncertainty." The presented optimal feedforward control model indeed supports this conclusion. However, a short discussion on whether this is the sole reason for co-contraction in uncertain environments might be beneficial. The authors' 2020 SOOC paper is a good example; it provided an alternative explanation for some behavioral observations that were previously modeled differently, e.g., the minimal intervention principle. Could there be alternative explanations for co-contraction yet to be discovered (maybe a feedback-driven one)?

We have expanded our analysis in the Discussion to address this interesting point about feedback-driven co-contraction, see lines 333-349: *"Although this work focuses on*

anticipatory co-contraction modeled as a feedforward mechanism, feedback-mediated co-contraction might also play a role [11]. Optimal feedback control typically corrects errors through reciprocal muscle activations; hence combining USOOC with a LQG layer, as discussed earlier, should result in increased co-contraction alongside reciprocal muscle responses to unexpected disturbances. This would align well with experimental observations showing that voluntary co-contraction is accompanied by reciprocal muscle responses to mechanical perturbations [7,11]. Feedback responses may nonetheless be modulated by co-contraction."

Regarding the reasons for co-contraction, we have decided to emphasize that co-contraction can also result from the uncertainty about the initial/current state estimate (which is another source of internal uncertainty and which is central to high-level feedback loops). We believe that this point could be crucial to command co-contraction in a receding horizon control scheme based on the USOOC framework (see lines 319-327).

Lastly, the manuscript is well-written, though a round of copy-editing could enhance its clarity and impact.

As suggested, we have carefully reread the manuscript.

Here are some additional comments

Line 63. Problem 1. The dynamics of the nonlinear model must be approximated (linearized) to arrive at this augmented-state ODE description. However, the Discussion (lines 253-254) tries to contrast the model with "linear" theories like H-infinity. Further discussion is appreciated to expand on when and how the presented method can be applied to full nonlinear system equations (i.e., without approximation), or how the approximation is still superior to other methods.

Thank you for this question. It is important to note that the augmented deterministic optimal control problem retains the nonlinear nature of the neuromusculoskeletal system in term of the propagation of the mean state. Considering the nonlinearity of the system is essential as it allows the control variable u to influence the covariance propagation as well. The real linearization (i.e., involving the drift's Jacobian) only appears in the equation for covariance propagation. This is an approximation for nonlinear drifts (except for bilinear ones) but it is sufficient to capture how the open-loop control u can modulate the covariance of the state x. We note that starting with a linear drift (function f) would not allow us to make any meaningful predictions about feedforward impedance control; bilinear drifts are the simplest case where such predictions become relevant. In contrast, H-infinity control assumes a linear drift, so that it is only thanks to state feedback that it can change the covariance of the state x. Therefore, any linear control system theory (very popular in motor control studies) will fail to capture the nonlinearities of the neuromusculoskeletal system that are essential for eliciting feedforward co-contraction and in turn impedance control. We have clarified these points at lines 284-294 and 359-361 and in the remark at lines 407-413.

Equations 6, 7, 10, and 25 need parentheses in the integral.

We have added parentheses.

115. How was the multiplicative noise included in the model? Please provide more details.

We have added more details in the caption of Fig. 1., which now defines precisely the diffusion term that we included.

Fig 1C. Why do the grey traces have two separate values for a given variance? In other words, why isn't the shape shown in Fig 1A symmetric? Is it because of some combined effects of intrinsic and extrinsic noise?

Thank you for pointing out this. We added an explanation at lines 132-140 for this asymmetry with multiplicative noise. The main reason is that the amount of noise scales with the optimal value of f:
"Interestingly, if we add a multiplicative noise in these simulations (10% of the input u), the above relationships remain valid but a lower stiffness becomes optimal in general. Indeed, a too large stiffness would increase uncertainty and the expected cost so that it is better to lower the overall stiffness in this case. However, it is worth noting that symmetry is broken with multiplicative noise because the amount of noise scales with f. For example, while both $\alpha = 0$ and $\alpha = 1$ do not introduce external uncertainty, the difference lies in the fact that with $\alpha = 1$, we have $f = -1$, which adds internal noise, whereas with $\alpha = 0$, $f = 0$ thus no additional noise occurs. Therefore it is optimal to slightly increase system stiffness to mitigate this internal motor noise when $\alpha = 1$."

120. A brief description of the model is appreciated here. Is it a linear or nonlinear model? How were stiffness and muscle activity related? I suggest including the equations of motion in the Methods section.

We have added a description of the nonlinear model in the Methods section at lines 551-558. Even simple muscle models with activation-dependent viscoelasticity are nonlinear. They are bilinear in the simplest case (see Eq. 27), with a muscle torque involving products between state and control elements. We also explained at lines 364-367 why it is crucial to consider nonlinear drifts to make meaningful predictions.

136. Fig 3 or Fig 4?

Thank you this is now corrected, where we now refer to both figures.

141. Please provide more details of this model as well.

We have added further details in the Methods section (see lines 566-594). However, we chose not to include exhaustive details within the paper to avoid excessive length. Since the full code is provided alongside the paper, all parameters and equations are readily accessible for any motivated reader who wishes to explore them in depth.

150. Torques are mentioned, but there are 3 traces in Fig 3C,D, which implies muscle groups. Please provide further details to clarify.

We apologize for the inaccuracies in the original description. We have corrected the paragraph and provided additional details on lines 180-181. The captions of Fig. 3 and Fig. 4 have also been clarified to better explain muscle groups and the plots.

151. In the statement “mean stiffness and co-contraction”: Mean over time and over muscles? Further details are appreciated.

The reviewer is correct. We have added the following clarification (lines 181-182): *“For this planar arm reaching task, the evolution of mean stiffness/co-contraction (averaged across joints/muscles and over time) as a function of probability and task uncertainty is displayed in Figure 4.”.*

Figure 3C. It is curious that co-contraction is zero in the first quarter of movement. Is it just an artifact of the combination of reach direction, initial condition, disturbance magnitude, etc. in the simulation? Or is there some exciting explanation behind it?

In these simulations, the initial state uncertainty was set very low, which likely explains the absence of significant co-contraction at the start. When we ran simulations with a larger initial state covariance P_0, we observed an increase in co-contraction during the initial phase of motion. This adjustment and its impact on the control strategy are now discussed in the Results section at lines 178-179. An illustration of this control strategy is provided below.

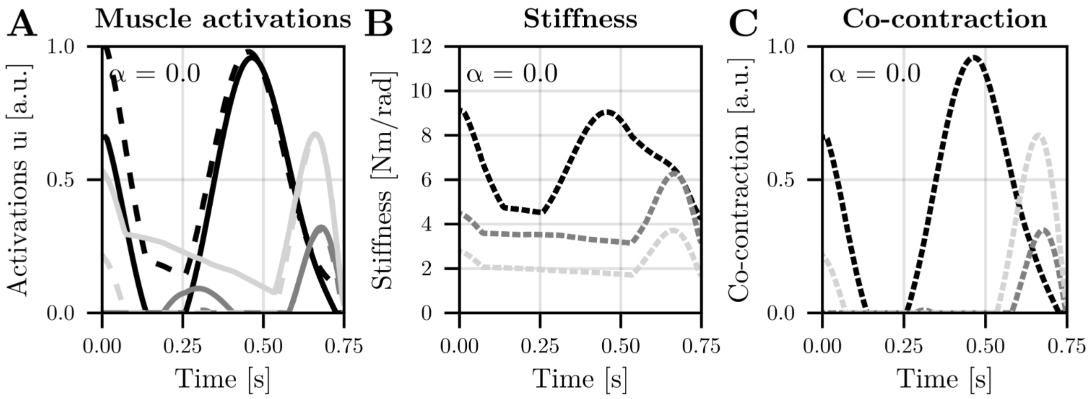

165. Was the disturbance always in the flexion direction (or extension)? Also, when describing the experimental protocol, it is mentioned in line 410 both flexion and extension movements, but the results (and line 421) imply only flexion movements were tested. The description of the results and experimental protocol require further expansion and clarification.

We agree that our initial description of the task was not sufficiently clear. We have now clarified at lines 496-497 that only flexion movements were perturbed, and detailed the direction of the perturbation.

303. Why are A, B assumed to be functions of u? Is it, for example to include muscle activation-dependent dynamics? What would be the applications/implications of this assumption?

The reviewer is correct, the notations A(u) and B(u) are to include the activation-dependent muscle dynamics. We have added this explanation at lines 364-367: *“This dependency on u can be used to consider bilinear drifts, corresponding to the simplest mechanical model for obtaining meaningful predictions about the planning of mechanical impedance (e.g., as illustrated in the toy example).”*

It is important to note that the restriction to deterministic, open-loop controls is crucial here to ensure that the corresponding equation represents an affine SDE (even though its deterministic counterpart, using the drift alone, would be classified as a nonlinear –precisely bilinear– system in classical control theory). We added a note at lines 359-361 to clarify this distinction. Additionally, we have expanded on this point in the Methods section (lines 407-413): *"Remark. Although referred to as statistical linearization, it is worth noting that the propagation of the mean retains the nonlinear characteristics of the drift (i.e., the deterministic dynamics). The linearized drift around the mean trajectory is primarily used to approximate the propagation of the covariance. This approximation is generally valid as long as the actual trajectories do not significantly deviate from the mean. In practice, this aligns with the goal of open-loop control (and, more broadly, feedback control) to reduce variance, thereby justifying the approximation. For a more rigorous treatment of these approximations, see [77]."*
