# Supplementary material for: Co-contraction embodies uncertainty: An optimal feedforward strategy for robust motor control
Source: PLoS Comput Biol. 2024 Nov 20;20(11):e1012598. doi: 10.1371/journal.pcbi.1012598 (PMC11616891; doi:10.1371/journal.pcbi.1012598)
Supplement: Attachment — Submitted filename: Re-review Berret 2024.pdf [file pcbi.1012598.s004.pdf]

Re-review of "Co-Contraction Embodies Uncertainty: An Optimal Feedforward Strategy for Robust Motor Control" by Berret et al., PCOMPBIOL-D-24-01022_R1

This revised paper has made a reasonable response to my previous concerns. The following minor suggestions would improve the manuscript.

The terms 'co-activation' and 'co-contraction' appear to be used interchangeably. If a distinction is intended, it would be well to explain that, perhaps in a footnote, on first use. Otherwise it would be better to consistently use one term.

The response to my concern about the apparently fast learning is reasonable, but it would be good to add this explanation to the main text, e.g. some version of "In the 100% condition, which preceded the α={0.25, 0.50, 0.75} conditions, participants required an average of about 50 trials to adapt and achieve a success rate above 80%. After this initial adaptation, participants indeed needed approximately 20 trials to adjust to the uncertain conditions."

The caption of Figure 2 refers to u_1 and u_2 as torques when apparently they are muscle activations (consistent with "arbitrary units").

In the caption of Figure 4 it would be helpful to spell out that these data are for the planar arm reaching task.

Copy editing, ideally by a native English speaker, is advised. For example, the opening sentence of the Abstract would read better as

"Despite our environment ~~is~~ often being uncertain, we generally manage to generate stable motor behaviors."

Line 23: "~~If~~ Although most computational models focus on the uncertainty arising from within the CNS …"

In many places singular and plural forms do not match, e.g. lines 53, 61, 90, 91, 95 … and many more.

Line 359 seems to imply that "affine" is linear (by contrast with non-linear); not so.
