# Supplementary material for: Co-contraction embodies uncertainty: An optimal feedforward strategy for robust motor control
Source: PLoS Comput Biol. 2024 Nov 20;20(11):e1012598. doi: 10.1371/journal.pcbi.1012598 (PMC11616891; doi:10.1371/journal.pcbi.1012598)
Supplement: Attachment — Submitted filename: response_letter_rev_final.pdf [file pcbi.1012598.s005.pdf]

We thank the Reviewers and the Editor for their feedback that helped us to improve the clarity of our paper. Below, we address each comment individually, with our responses highlighted in blue.

Reviewer #1

This revised paper has made a reasonable response to my previous concerns.

The following minor suggestions would improve the manuscript. The terms 'co-activation' and 'co-contraction' appear to be used interchangeably. If a distinction is intended, it would be well to explain that, perhaps in a footnote, on first use. Otherwise it would be better to consistently use one term.

We thank the reviewer for raising this point. To clarify this aspect, we added: "*Following [Burdet2013], we define coactivation as the minimum of the activities of antagonist muscles spanning a joint, and co-contraction as their sum*". The definition for the activities of an antagonist muscles pair is also clarified in the caption of Figure 2. The terms have been adjusted to ensure consistency throughout the paper.

The response to my concern about the apparently fast learning is reasonable, but it would be good to add this explanation to the main text, e.g. some version of "In the 100% condition, which preceded the α={0.25, 0.50, 0.75} conditions, participants required an average of about 50 trials to adapt and achieve a success rate above 80%. After this initial adaptation, participants indeed needed approximately 20 trials to adjust to the uncertain conditions."

We have incorporated this information into the text: "*In the 100% condition, which preceded the α={0.25,0.50,0.75} conditions, participants required about 30-50 trials to adapt and consistently achieve a success rate above 80%. After this initial adaptation, participants needed about 10-20 trials to adjust to the uncertain conditions*".

The caption of Figure 2 refers to u1 and u2 as torques when apparently they are muscle activations (consistent with "arbitrary units").

Indeed, we should have referred to muscle activations. This has been corrected in the revised version.

In the caption of Figure 4 it would be helpful to spell out that these data are for the planar arm reaching task.

We added the following information to the caption: "*Evolution of the optimal stiffness and coactivation for the planar arm reaching task under uncertainty*"

Copy editing, ideally by a native English speaker, is advised. For example, the opening sentence of the Abstract would read better as "Despite our environment is often being uncertain, we generally manage to generate stable motor behaviors." Line 23: "If Although most computational models focus on the uncertainty arising from within the CNS ..." In

many places singular and plural forms do not match, e.g. lines 53, 61, 90, 91, 95 ... and many more.

We have corrected the wording and resolved the mismatched singular/plural forms. Additionally, we reviewed the entire paper to correct any English errors.

Line 359 seems to imply that “affine” is linear (by contrast with non-linear); not so.

We have revised the sentence as follows: “*We first focus on the affine case, then on the general nonlinear case*”.
